# Use of anakinra in cryopyrin-associated periodic syndromes: case report and review of literature

**DOI:** 10.3389/fimmu.2025.1591234

**Published:** 2025-06-05

**Authors:** Marta Salas Sánchez, Elena García-Martínez, Héctor Balastegui Martín, María Esther Durán-García, Cristina Lavilla Olleros

**Affiliations:** ^1^ Department of Internal Medicine, Hospital General Universitario Gregorio Marañón, Madrid, Spain; ^2^ Immunology Department, Hospital General Universitario Gregorio Marañón, Madrid, Spain; ^3^ Instituto de Investigación Sanitaria Gregorio Marañón, Madrid, Spain; ^4^ Primary Immunodeficiencies Unit, Hospital Universitario y Politécnico La Fe, Valencia, Spain; ^5^ Pharmacy Department, Hospital General Universitario Gregorio Marañó, Madrid, Spain

**Keywords:** cryopyrin-associated periodic syndrome, CAPS, autoinflammatory disease, anakinra, case report

## Abstract

Cryopyrin-associated periodic syndrome is a rare autoinflammatory syndrome caused by a dysregulation of cytokine signaling pathways, particularly of the interleukin-1β production. Symptoms typical appear early in life and include recurrent fever, arthralgia, and cutaneous rash, often accompanied by systemic manifestations and progressive end-organ damage resulting from chronic inflammation. Interleukin- targeted therapy constitutes the basis for treatment, and can lead to complete resolution of symptoms. We report a case of a 66-year-old man with cryopyrin-associated periodic syndrome related to V198M mutation, successfully treated with anakinra, an interleukin- 1 inhibitor frequently used in rheumatoid arthritis.

## Introduction

Cryopyrin-associated periodic syndromes (CAPS) are a group of rare hereditary autoinflammatory diseases caused by an overproduction and excessive activation of interleukin-1β, which, in turn, results from gain-of-function mutations in the NLRP3 gene (1). Other autoinflammatory diseases include Familial Mediterranean fever (FMF) and tumor necrosis factor receptor-associated periodic syndrome (TRAPS), and although they share some common features with autoimmune diseases, the physio- pathological mechanisms behind these entities are distinct (2). In autoinflammatory diseases, symptomatology results from dysregulation of pathways associated with the innate immune response, such as inflammasome and NF-κB activation disorders or cytokine response dysregulation. In contrast, in autoimmune diseases, it results from the presence of autoreactive B and T lymphocytes (2). From a clinical point of view, CAPS’ hallmark triad consists of fever, arthralgia, and cutaneous rash, however systemic involvement, including that of the central nervous system, is possible, especially in severe cases (3). Nowadays, treatment is based on interleukin- targeted therapy, constituted by three main drugs: anakinra, rilonacept and canakinumab (4).

Herein, we report the case of a 66-year old man with a diagnosis of a mild variant of CAPS, with a missense mutation in the NLPR3 gene, successfully treated with anakinra.

## Case presentation

A 66-year-old man with a history of arterial hypertension, chronic ischemic heart disease, and secondary chronic kidney disease presents with recurrent episodes of fever. These episodes were accompanied by arthralgia, predominantly affecting large joints, and occasionally by an urticaria-like rash mainly involving the upper limbs. Symptoms had reportedly begun during childhood, were self-limited lasting approximately 24 to 72 hours, and had no identifiable triggering factors. He reported between 2 to 3 episodes a year.

On physical examination, the patient was febrile (temperature 38.5°C) but appeared otherwise well. He did not present with a cutaneous rash, or any other pathological findings.

Laboratory evaluations were notable for mild normocytic anemia and elevated inflammatory markers (C-reactive protein 26 mg/dL; erythrocyte sedimentation rate 85 mm/h; ferritin 1160 μg/L). Serum amyloid was unavailable at the time. Autoimmune screening revealed no abnormalities, and quantitative serum immunoglobulin measurements (IgA, IgM, IgG, and IgG4 levels) were all within normal limits. All serologic testing performed was negative. A summary of the laboratory findings is provided in **Tables 1**, **2**.

**Table 1:** Laboratory data at initial evaluation.

Parameter (units)	Value	Normal range
WBC (x10^3^ μl)	13.30	4.0 - 10.0
Hemoglobin (g/dL)	12.3	13.0 - 17.5
MCV (fL)	84.0	80.0 – 98.0
Platelets (x10^3^ μl)	312	140 - 400
Creatinine (mg/dL)	1.37	0.7- 1.20
GFR (ml/min/1.73m^2^)	52	>60
ESR (mm/h)	66	2 - 14
C-reactive protein (mg/dL)	26.6	22 – 274
Ferritin (μg/L)	1160	0.00 - 15.00
ANA	Negative	
ANCA (P-ANCA, C-ANCA, A-ANCA)	Negative	
Anti-ds DNA (UI/mL)	0.50	0.0 – 15.0
Rheumatoid factor (UI/mL)	<16.5	0.0 – 25.0
C3 (mg/dL)	133.0	85.0 – 177.0
C4 (mg/dL)	44.1	17.0 – 52.0
IgA (mg/dL)	255.0	103.0 – 568.0
IgM (mg/dL)	112.0	38.0 – 231.0
IgG (mg/dL)	1130.0	680.0 – 1670.0
IgG4 (g/L)	0.14	0.032 – 1.315

ANA, antinuclear antibody; ANCA, anti- neutrophil cytoplasmic antibody; ESR, erythrocyte sedimentation rate; GFR, glomerular filtration rate; MVC, Mean corpuscular volume; WBC, white blood cell.

**Table 2:** Summary of microbiological and serological workup.

Test	Result
Hepatitis C (anti-HCV CLIA)	Negative
Hepatitis B (anti-HBc IgM and IgG)	Negative
HIV (antigen/antibody)	Negative
EBV IgM	Negative
CMV IgM	Negative
Parvovirus B19 IgM	Negative
Toxoplasma IgM	Negative
Syphilis screening	Negative

CMV, Cytomegalovirus; EBV, Epstein–Barr virus.

Additionally, a Positron Emission Tomography/Computed Tomography (PET-TC) scan was performed, without any pathological findings. Given the suspected diagnosis of an autoinflammatory disease, the patient underwent molecular genetic testing, which reported a well-known heterozygous missense mutation in the gene that encodes the NLPR3 protein: NM_001243133.2:c.592G>1; p.Val198Met. These findings did not allow for a genetic diagnosis of CAPS. However, despite the non-conclusive molecular results, the clinical history and the exclusion of other conditions supported a clinical diagnosis.

Treatment with anakinra 100mg subcutaneous daily was initiated, achieving clinical remission within the first month of treatment and drastic improvement of symptomatology within days. Initial score for autoinflammatory disease activity index (AIDAI) and Physician Global Assessment (PGA) was of 81 and 3 respectively, reaching a score of 4 and 0 respectively after 10 days of initiating treatment. Inflammatory markers, including C-reactive protein, erythrocyte sedimentation rate, and ferritin, which had been markedly elevated during febrile episodes and mildly elevated between flares, normalized within 30 days of initiating treatment.

After 24 months of continuous treatment, the patient remained well, with no new flares, and no relevant adverse effects. A trial of on-demand therapy during flares lasting at least seven days was initiated, based on the patient’s clinical improvement and inconclusive genetic findings. This strategy followed a similar approach used in other monogenic autoinflammatory disorders, such as FMF.

This change in strategy resulted in multiple outbreaks (AIDAI 72, Physician Global Assessment PGA 3), leading to the permanent reintroduction of treatment, initially with a spaced therapeutic regime (anakinra 100mg administered every 48h), and eventually a conventional regime (anakinra 100mg administered daily). The patient reported no further relevant outbreaks after, remaining asymptomatic with excellent tolerance to the drug after 6 months of treatment.

**Figure 1:**
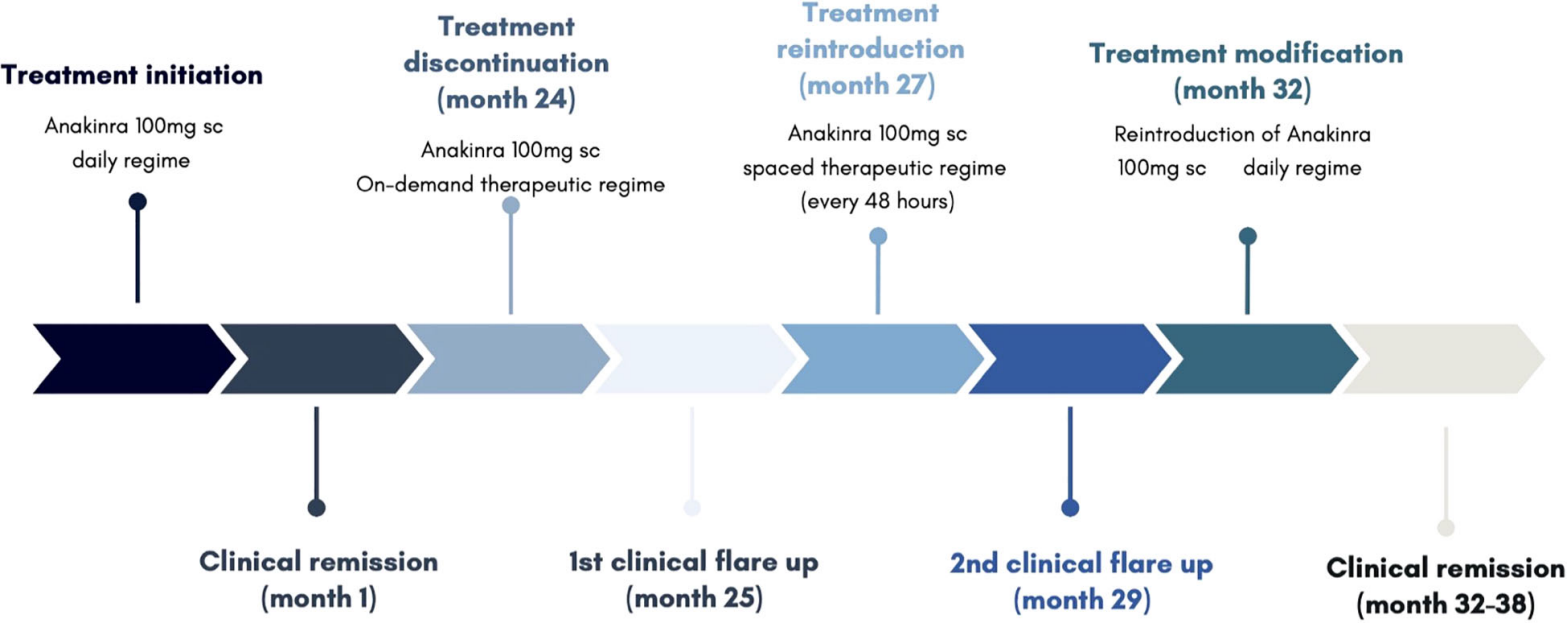
Clinical and therapeutic course.

The clinical manifestations and therapeutic interventions of the patient have been summarized in **Figure 1**.

## Discussion

Cryopyrin-associated periodic syndromes are caused by gain-of-function mutations in NLRP3 gene, encoding for cryopyrin, which constitutes a key component of the NLRP3 inflammasome. These mutations ultimately lead to an inflammasome over-activation that in turn leads to an uncontrolled release of interleukin-1β, prompting chronic systemic inflammation (1). The term “cryopyrin-associated periodic syndromes” refers to a group of entities which all form part of a clinical continuum of a single disorder, sharing several characteristics but differing in severity. Within this spectrum, three main clinical entities have been identified, ordered from least to most severe: familial cold autoinflammatory syndrome (FCAS), Muckle-Wells syndrome (MWS), and neonatal- onset multisystem inflammatory disease (NOMID), also known as Chronic Infantile Neurologic Cutaneous Articular Syndrome (CINCA) (4).

Clinically, these disorders are characterized by recurrent episodes of fever, arthralgia, urticaria-like cutaneous rash, and ocular inflammation, occurring in the absence of apparent causes, including infectious, neoplastic, and/or autoimmune conditions. Additionally, there may be involvement of the central nervous system and sensorineural hearing loss in the more severe cases, as well as secondary amyloidosis as result of chronic inflammation (5). Diagnosis requires a high index of suspicion, and is based on compatible clinical findings. Molecular testing may aid in confirming a diagnosis in those patients presenting with a consistent clinical phenotype. However, congruent genetic findings alone, without compatible clinical manifestations, are insufficient to establish a diagnosis and do not warrant initiation of treatment. It is also worth noting that a genetic analysis is not strictly necessary for diagnosis, and that lack of abnormal genetic testing does not exclude diagnosis in any case (6).

In our patient’s case, the clinical signs were quite indicative, while the genetic findings were less clear, as molecular genetic testing disclosed a well-known but inconclusive heterozygous missense mutation in NLRP3 gene (NM_001243133.2:c.592G>1; p.Val198Met). This protein modification has been considered as a conflictive variant with different interpretations of pathogenicity as it has been found both in CAPS’ patients, as well as in unaffected relatives and asymptomatic controls (highest allele frequency of 0.0238 in European-finish population) (7).

Rowczenio et al. investigated the NLRP*3* gene in 830 individuals at a UK specialist center. The cohort consisted primarily of patients with suspected autoinflammatory disease (90%), along with family members of individuals previously identified as carrying the V198M variant (10%). Among those screened, the V198M variant was detected in 19 individuals (2.4%), of whom only five were ultimately diagnosed with CAPS (**Table 3**). Three individuals were diagnosed with other autoinflammatory conditions, including TRAPS, Schnitzler syndrome, and mevalonate kinase deficiency (MKD), while five presented with nonspecific autoinflammatory syndromes. The remaining individuals remained asymptomatic.

**Table 3:** Phenotypes associated with NLRP3 V198M variant in CAPS.

Case	Sex	Age at presentation (years)	CAPS subtype	NLRP3 Mutation	Clinical features	Treatment/ dose	Response	Previous treatment/response	Reference
1	F	29	Unspecified	V198M	Arthralgias, myalgias, uveitis, abdominal pain, aphthous ulcera, migraine	Anakinra Dose N/A	N/A	GCs/N/A	Schuh et al. (9)
2	M	36	Unspecified	V198M	Recurrent urticarial rash, bursitis, tendinitis	None Dose N/A	N/A	None	Schuh et al. (9)
3	M	35	MWS	V198M + R260W (hetero-zygous)	Lymph node enlargement, ar- thralgia, chronic urticaria, intestinal and renal AA amyloidosis	Canakinumab 150mg s.c	Complete resolution of symptoms, improved but persistent renal impairment and proteinuria.	Colchicine/no Prednisone, cyclophosphamide/partial response	Scarpioni et al. (10)
4	M	12	MWS	V198M + R260W (hetero-zygous)	Periodic fever, arthralgia, urticarial rash, renal AA amyloidosis	Canakinumab 150mg s.c	Complete resolution of symptoms, improved but persistent renal impairment and proteinuria.	Colchicine/no	Scarpioni et al. (10)
5	N/A	Neonatal	MWS Overlaping features with FCAS and CINCA/ NOMID	V198M	Urticarial rash, arthralgia, hearing impairment, dysmorphic features (short stature, frontal bossing of skull, flattening of nasal bridge)	Anakinra Dose N/A	Complete	N/A	Rowczenio et al. (8)
6	N/A	Neonatal		V198M		Anakinra Dose N/A	Complete	N/A	Rowczenio et al. (8)
7	N/A	Neonatal		V198M		Anakinra Dose N/A	Complete	N/A	Rowczenio et al. (8)
8	N/A	2	MWS	V198M	Periodic fever, urticarial rash, arthralgia	N/A	N/A	N/A	Rowczenio et al. (8)
9	N/A	4-13	MWS	V198M	Pyrexia, conjunctivitis, headache, abdominal pain, arthralgia and lassitude	Canakinumab 2 mg/kg s.c.	Complete, however required rescue dose and/or dosage increase	Anakinra Partial response, frequent necessity of dosage increase	Kuemmerle-Deschner et al. (11)
10	N/A	4-13	MWS	V198M	Pyrexia, sensitivity to infection, coldness exposed exanthema, conjunctivitis, headache, oral aphthae, abdominal pain, myalgia and fatigue.	Canakinumab 2 mg/kg s.c.	Complete, however required rescue dose and/or dosage increase	Anakinra Partial response, frequent necessity of dosage increase	Kuemmerle-Deschner et al. (11)
11	M	9	MWS	V198M	N/A	Canakinumab 2mg/kg s.c.	Complete, however required high doses (10mg/kg i.v)	Anakinra Partial response	Hansmann et al. (12)

CINCA, Chronic Infantile Neurologic Cutaneous Articular Syndrome; FCAS, Familial Cold Autoinflammatory Syndrome; GCs, Glucocorticoids; MWS Muckle-Wells Syndrome; N/A Not Available; NOMID Neonatal-Onset Multisystem Inflammatory Disease.

More recently, V198M has been reclassified as a low-penetrance allele potentially associated with variable, often atypical, presentations of CAPS, with frequent overlap features of MWS, FCAS, and NOMID/CINCA (7–12). It is most frequently linked to milder phenotypes, typically with late onset and non-specific symptoms such as fatigue and malaise (8). However, it has also been associated with variable responses to anti-IL1 therapy, with some patients requiring higher doses or more frequent administration to sustain therapeutic effectiveness (**Table 3**) (11).

Recently, a diagnostic model has been proposed, which focuses primarily on clinical data. It requires the presence of raised inflammatory markers (C reactive protein: CRP and/or serum amyloid A: SAA), and at least two of six typical signs and/or symptoms of CAPS: urticarial-like rash, cold/stress triggered episodes, sensorineural hearing loss, musculoskeletal symptoms (arthralgia, arthritis/myalgia), chronic aseptic meningitis and/or skeletal abnormalities (epiphyseal overgrowth/frontal bossing). With a sensitivity of 81% and a specificity of 94%, it allows for an adequate diagnosis of CAPS and its different subtypes, both in the presence and absence of genetic findings, including conflictive variant with different interpretations of pathogenicity (13). Its main components are summarized in **Table 4**.

**Table 4:** Adapted from Kuemmerle-Deschner et al.

Proposed diagnosis model for CAPS
Raised inflammatory markers (CRP/SAA)
Two or more out of six typical signs/symptoms (1)Urticaria-like rash (2)Cold/stress triggered episodes (3)Sensorineural hearing loss (4)Musculoskeletal symptoms: arthralgia, arthritis, myalgia. (5)Chronic aseptic meningitis (6) Skeletal abnormalities: epiphyseal overgrowth, frontal bossing.

CRP, C- reactive protein; SAA, serum amyloid A.

Disease activity in CAPS, as well as other autoinflammatory disorders, is assessed using the validated autoinflammatory disease activity index (AIDAI). This index contains 12 items including fever (>38°C), overall symptoms and organ-specific AID symptoms, scored as 1 (present/yes) or 0 (absent/no). The maximum daily score is 12 with an accumulative monthly score raging from 0 to 372 (31-day month). Inactive disease is defined as an AIDAI score below 9/month. Physician Global Assessment (PGA) constitutes another useful surveillance tool, mainly used for therapeutic drug monitoring (TDM) (7).

Regarding treatment, the identification of NLRP3 gene mutations and the growing understanding of inflammatory cytokine activation pathways, particularly IL-1β, have led to the development of highly effective therapies.

There are currently three anti-interleukin-1 drugs: anakinra, canakinumab and rilonacept (14, 15). Among these therapies, anakinra and canakinumab are the most commonly used. Both are interleukin-1 antagonists, and have shown effectiveness in terms of symptom remission and improvement of quality of life of patients, however they present differences in their pharmacological profiles and modes of administration.

Anakinra, which is administered daily via subcutaneous injection, effectively reduces inflammation and controls symptoms in real time (14, 16). Its use, however, requires careful monitoring of the patient’s clinical response. Its safety profile is generally favorable, with limited, manageable side effects. Common adverse events include injection site reactions and a slightly elevated risk of infections, similar to other biological treatments. It is worth noting that, in some cases, extending the dosing interval may be an option, particularly for patients experiencing mild side effects while maintaining adequate symptom control (14, 16). Regular monitoring and risk assessment are crucial to optimizing its use in managing CAPS.

Canakinumab, administered at less frequent intervals (typically every 4 to 8 weeks), may provide greater convenience for patients by minimizing the frequency of injections (14). However, a notable drawback of canakinumab compared to anakinra is its higher cost, which could limit accessibility for some patients within our healthcare system.

In summary, anakinra is preferred initially due to its rapid efficacy and accessibility, while canakinumab is reserved for cases where treatment with anakinra is inappropriate. In our patient’s case, treatment with anakinra was selected mainly due to its greater accessibility in our healthcare system.

A particularly noteworthy aspect of this case is that, despite the absence of a definitive molecular diagnosis, the patient experienced clinically significant symptoms that deeply impacted his quality of life, and could potentially lead to long-term complications, such as secondary amyloidosis. This justified the decision to initiate therapy. The marked clinical improvement, along with symptom recurrence following temporary treatment discontinuation, further reinforced our clinical suspicion and supported the decision to continue therapy. This case underscores the complexities of managing CAPS and related autoinflammatory disorders, particularly in patients with inconclusive genetic findings, highlighting the value of a symptom-driven therapeutic approach in ‘grey zone’ cases. Moreover, the recurrence of symptoms following treatment withdrawal emphasizes the challenges of tapering therapy, even in apparent remission, and offers practical insights for long-term management decisions.

A notable limitation of this case is the inability to perform genetic studies on the patient’s parents or offspring, which impedes determining whether the mutation is *de novo* or inherited, as well as the potential presence of epigenetic variations.

## Conclusion

Advances in the understanding of the pathophysiological basis of cryopyrin-associated periodic syndromes, have led to significant improvements in diagnosis and treatment. In the absence of definitive genetic confirmation, a clinical diagnosis may be considered appropriate in the presence of a highly suggestive phenotype and a favorable therapeutic response. Anakinra appears to be a safe and effective drug, significantly contributing to symptom control and improving the quality of life for patients with this complex disease. Further research is needed to determine the optimal duration of treatment, potential long-term adverse effects, and the importance of evaluating not only clinical efficacy but also the economic impact when selecting a treatment approach. Multidisciplinary management, including specialties such as Internal Medicine, Rheumatology, Immunology and Clinical Pharmacy, is essential.

## Data Availability

The original contributions presented in the study are included in the article/supplementary material. Further inquiries can be directed to the corresponding author.
